# From risky individuals to risky systems: a conceptual framework for the next generation of forensic mental health research

**DOI:** 10.3389/fpsyt.2025.1739679

**Published:** 2025-12-16

**Authors:** Anne G. Crocker, Marichelle Leclair

**Affiliations:** 1 Department of Psychiatry and Addictions & School of Criminology, Université de Montréal, Montréal, QC, Canada; 2 Institut National de Psychiatrie Légale Philippe-Pinel, Montréal, QC, Canada; 3 Department of Psychoeducation and Psychology, Université du Québec en Outaouais, Gatineau, QC, Canada

**Keywords:** forensic mental health services, violence risk, mentally disordered offenders, policy, assessment, ecological model, risk-needs-responsivity, resilience

## Abstract

Risk in forensic mental health is often shaped as an individual issue. But what if risk is also produced by the systems meant to provide care and safety? This paper introduces the *Risk in Systems Framework*, a conceptual model to understand how risk emerges not just within people, but also through institutional practices and structural inequalities. Drawing on established models from criminology, psychology, and public health, the framework explores risk across three levels: the individual, the system, and the broader social structures. It helps identify how policies, professional norms, and historical legacies can shape who is labelled risky and what responses are considered appropriate. By shifting the focus from “risky individuals” to “risky systems,” this approach supports more responsive care for people in forensic mental health settings.

## Introduction

Forensic mental health is an area of research, care and policy that the public most often hears about in the wake of tragedy – usually when a person living with severe mental illness is the perpetrator of a violent offense. These moments prompt intense debate about the association between mental illness and violence, the adequacy of public safety, and the provision of evidence-informed care (1, 2). Yet examples from around the world show that political responses often take the form of knee-jerk investments in control policies and public safety infrastructures, despite a lack of evidence for their effectiveness (3, 4). This is frequently done at the expense of upstream strategies: prevention programs, community and family support, opportunities for social and economic participation, and the broader work of reducing marginalization and poverty.

As of 2025, forensic mental health systems remain structured around individualized, deficit-focused models shaped by colonial logics, that is, institutional norms rooted in Western notions of knowledge, expertise, and authority. These frameworks pathologize, marginalize, and punish, leaving unexamined the structural conditions that shape both risk and recovery. Do our systems set people up for failure?

In this paper, we call for a fundamental reorientation of forensic mental health research: shifting our attention toward systemic resilience, contextualized care, prevention, and community-grounded support. To ground this proposal, we first analyze the historical and conceptual legacy that shaped the field. Then, drawing from Andrews and Bonta’s Risk-Needs-Responsivity model (5, 6), Bronfenbrenner’s ecological systems theory (7), and Haldane et al.’s Health Systems Resilience Framework (8), we propose a new conceptual framework centered on systemic accountability. By opening new research avenues and reframing the knowledge base, we aim to inform practice and policy in ways that address both system-level risks and opportunities alongside individual-level needs, ultimately contributing to the prevention of violence and criminal justice involvement among people living with mental illness.

### Historical and conceptual legacy that shaped the field

Over the past several decades, forensic mental health research has developed into a dense and expanding field. Early studies focused on epidemiological links between mental illness and violence (9–12), followed by the development of typologies to categorize individuals who exhibit violent or criminal behavior (13–15). In parallel, risk assessment tools and psychometric research proliferated (16–20) and quickly became central to both clinical and policy discourse (21, 22). Subsequent waves of scholarship have explored the effects of stigma (23–27), examined personal recovery processes (28–32), and more recently, described emerging models of care (33, 34) and the use of new technologies in managing violence risk (35, 36).

To trace the evolution of these research priorities, we conducted a rapid bibliometric time-trend analysis of the top 10% most-cited papers – an imperfect but useful proxy for influence. Using the Web of Science database and the Bibliometrix R package (37), we analyzed the 1,155 most cited publications in the field. This analysis reveals a striking concentration of influence among literature produced in high-income, English-speaking countries. The United States alone accounted for more than 46,000 citations (based on first author’s affiliation), followed by Canada (14,607), the United Kingdom (11,738), Australia (4,858), and Germany (4,264). This concentration of influence likely reflects several structural factors: English is the lingua franca of scientific publishing (giving English-speaking institutions an inherent advantage), most major journals and indexing systems are based in English-speaking countries, and these countries tend to have well-funded research infrastructures. Their forensic mental health systems also share historical lineages, which makes their scholarship more easily circulated and cited across these jurisdictions. These structural asymmetries in visibility, funding, and language can create the impression that the approaches developed in these countries represent a universal perspective on forensic mental health, even though they do not.

**Figure 1:**
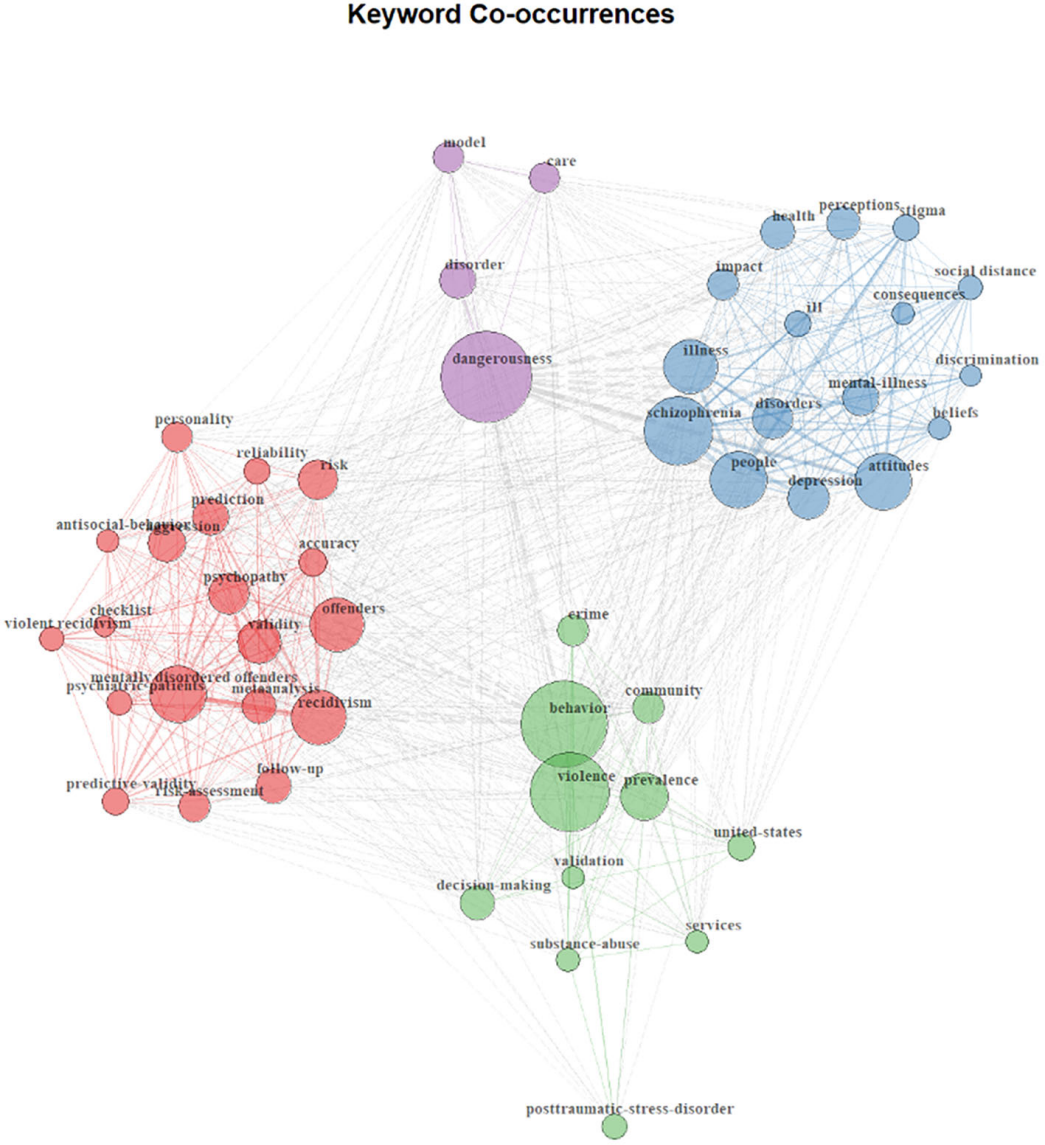
Conceptual landscape of the most cited research in forensic mental health.

**Figure 1** depicts a keyword co-occurrence network of the 50 most-cited papers in forensic mental health. Each node represents a keyword, and the size of the node reflects how frequently that keyword appears in the dataset. Lines connecting nodes represent co-occurrences within the same paper; the thicker the line, the more often those keywords appear together. The colors indicate clusters of keywords that co-occur more frequently with one another than with other clusters, highlighting distinct conceptual groupings within the field.

The first (red) cluster centers on psychometric and actuarial research on risk assessment, with keywords such as *mentally disordered offenders, psychopathy*, *recidivism*, and *predictive validity*. A second (green) cluster orbits around *prevalence, behavior*, and *violence*, pointing to epidemiological work. A third (blue) is organized around *attitudes*, *stigma*, and *discrimination*, highlighting research that focuses on public perceptions and barriers faced by people with mental illness. Finally, a smaller cluster (purple) centers on *dangerousness*, and links to *mode*l and *care*. Together, these clusters outline the field’s conceptual terrain: stigma and perception studies remain largely siloed from dominant risk-assessment discourse, while structural and systemic perspectives are almost entirely absent.

## From risky individuals to risky systems

The bibliometric analysis shows that, despite decades of research, forensic mental health remains overwhelmingly focused on the individual: assessing their level of risk, diagnosing their deficits, and guiding their rehabilitation or containment. As a result, remarkably little is known about the systemic and structural factors that shape trajectories into, through, and beyond forensic systems. Few empirical studies, with some exceptions (38–42), have examined how policies, service design, institutional cultures, or broader sociopolitical forces contribute to violence or criminalization, or how these same forces affect recovery and reintegration.

There is a pressing need for a conceptual framework that extends beyond the individual to account for the interplay between personal, systemic, and structural factors. Such a framework could reposition responsibility not only on persons navigating these systems, but also on the societal and governmental decisions that shape their contexts. It could help shift the emphasis from managing risky individuals to interrogating risky systems; that is, systems that may reproduce stigma, exclusion, or punitive responses under the guise of care, thus creating risk within individuals.

### Foundational models

In this section, we begin to outline what such a framework might look like. We draw from three foundational models from different disciplines: the Risk-Need-Responsivity framework (5), the ecological systems theory (7), and health systems resilience (8). While each of these has its own limitations, together they offer a starting point for conceptualizing how risk and recovery are shaped not just by individual traits, but by the systems in which people live, and the histories and power relations that structure those systems.

#### Managing individuals: the risk-needs-responsivity model

The Risk-Need-Responsivity model remains the dominant framework in correctional services (6) and has gained traction in forensic mental health systems despite a limited evidence base (43–46). It organizes rehabilitation efforts around three principles (risk, need, and responsivity), all of which are rooted in individual-level assessment and intervention.

The Risk principle suggests matching the intensity of interventions to a person’s assessed risk of reoffending. The Need principle focuses on targeting an individual’s dynamic criminogenic needs such as antisocial attitudes, procriminal relationships, or substance use. Although mental health symptoms or trauma can, in some cases, influence recidivism and thus be conceptualized as dynamic needs, the framework has historically classified them as non-criminogenic. The Responsivity principle encourages adapting interventions to the individual’s strengths, learning styles, culture, and motivation. At its core, the Risk-Need-Responsivity framework remains centered on the individual and its immediate environment. The framework leaves little room for considering how factors like systemic racism, housing precarity, or punitive legal regimes create or maintain the very risks and needs it seeks to manage.

#### Contextualizing the individual risk: Bronfenbrenner’s ecological model

Bronfenbrenner’s ecological systems theory (7) offers a valuable lens to expand forensic mental health research beyond the individual and its immediate environment. Elaborated in developmental psychology, the model conceptualizes the individual as situated within a series of nested and interacting systems.

For our purposes, the model is useful for its capacity to situate individual risk and recovery within broader structural contexts. While the individual remains at the center, the model allows for a broader understanding of how behavior emerges through interactions with surrounding systems. The microsystem includes direct relationships and environments such as family, clinicians, and peers. These are shaped by the mesosystem, which reflects how these immediate systems relate to one another (e.g., coordination between care providers, or communication between providers and families). The exosystem brings into focus more distal structures that still exert influence, such as housing availability or policing. The macrosystem encompasses broader cultural and ideological forces, including policies, laws, dominant narratives about crime, laws, structural stigma, racism, and colonial legacies. Finally, the chronosystem introduces a temporal dimension, drawing attention to historical shifts and long-term impacts—such as policy reforms, changes in public discourse, and intergenerational trauma.

#### Shifting the focus: health systems resilience

Bronfenbrenner’s model reminds us that individual outcomes are shaped by systems – but which aspects of those systems matter, and how can they adapt? Public health offers valuable insight through health systems resilience: a system’s capacity to prepare for, absorb, and adapt to stress (47). Applied to forensic mental health, resilience reflects a system’s ability to deliver safe, equitable, and meaningful care under pressures such as resource scarcity, political shifts, and fear-driven narratives.

Drawing from the WHO’s health systems framework (48), Haldane and colleagues (8) identify five elements of resilience: governance and financing, workforce, service delivery, public health functions, and access to technologies. These are sustained through coordination across sectors and anchored in equity and community engagement. Each domain is relevant to forensic mental health. Governance and financing determine whether investment flows to secure institutions or to community-based housing, employment, and supports. Workforce considerations highlight the importance of safe staffing levels and developing a motivated, well-trained, culturally representative forensic workforce. Service delivery must be continuous, coordinated, and person-centered, yet remains fragmented in many systems. Public health functions (surveillance, early intervention, system-level monitoring) are underdeveloped, limiting our capacity to adapt. Finally, access to technologies, including digital tools and health information systems, is increasingly important yet unevenly distributed. Critically, centering community engagement means embedding people with lived experience, Indigenous peoples in settler-colonial contexts, and other local community partners into the design, governance, and evaluation of forensic systems.

## The risk in systems framework: a conceptual model to analyze and respond to personal, systemic, and structural risk in forensic mental health

**Figure 2:**
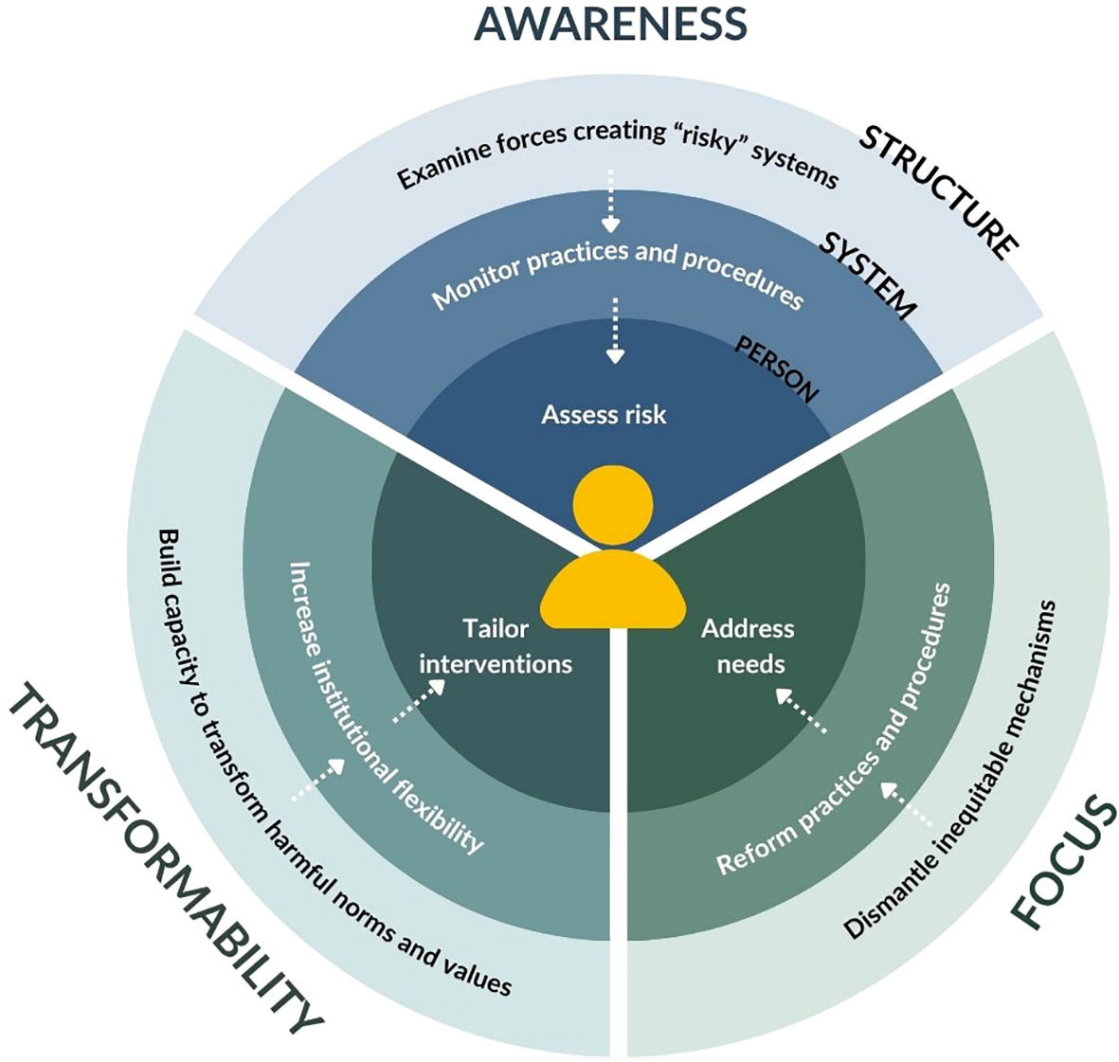
The risk in systems framework.

We build on these three complementary models – the Risk-Need-Responsivity framework, Bronfenbrenner’s ecological systems theory, and health systems resilience approach – to propose a conceptual framework that expands how risk is understood and studied in forensic mental health. **Figure 2** illustrates this framework across three interconnected levels (person, system, and structure), showing how each level aligns with the principles of awareness, focus, and transformability. These principles operate differently at each level, and the following section describes how they are operationalized in practice.

The original Risk-Need-Responsivity model (5) centers primarily on the individual and their immediate context. It involves *risk awareness* through assessing an individual’s likelihood of reoffending to calibrate intervention intensity (risk principle), *focus* by addressing criminogenic needs such as substance use or antisocial traits (need principle), and *transformability* through adapting interventions to the person’s learning style, culture, and identity to maximize engagement (responsivity principle). This layer is the person-level risk.

We build on this by proposing a first expansion: system-level risk (see **Figure 2**). While the individual’s risk remains the focus, this level acknowledges that risk is profoundly shaped by the institutions and systems surrounding them. Health, social, justice, and policing systems – including their structure, governance, and resource allocation – directly influence a person’s ability to access timely, appropriate care. These factors form part of the exosystem in Bronfenbrenner’s model and are key contributors to institutional and systemic risk. Drawing on the health systems resilience framework proposed by Haldane et al. (8), we can examine how risk is generated through breakdowns in governance, service delivery, workforce capacity, health information systems, financing, and community engagement. For example, insufficient staffing, poor continuity of care, and underrepresentation of marginalized communities in the workforce may erode trust and compromise safety. At this level, the principles of risk awareness, focus, and transformability that we derived from the Risk-Need-Responsivity model are operationalized as follow:

1. Risk awareness becomes monitoring how specific institutional practices, procedures, or mechanisms generate or exacerbate risk for individuals;
2. Focus refers to reforming institutional practices, procedures, or mechanisms that generate or exacerbate risk;
3. Transformability becomes increasing institutional flexibility to adjust practices to user needs and context.

A second, more transformative shift occurs when considering structure-level risk – the macrosystemic conditions that shape how health, social, and justice systems operate. Structural risk refers to the deep-rooted political, historical, and ideological forces, such as colonialism, racism, and neoliberalism, that influence how institutions are designed, resourced, and governed. These forces manifest in laws, policies, professional norms, and dominant assumptions about safety, control, and accountability. Forensic systems embedded in structurally unjust contexts may consistently reproduce inequity, even when frontline practices are well-intentioned. At this level, the three principles are again re-operationalized:

1. Risk awareness involves examining how ideologies, policies, and other structural forces create or amplify “risky systems”;
2. Focus becomes efforts to dismantle the structural mechanisms that produce systemic disadvantage;
3. Transformability reflects building structural capacity and willingness to transform dominant norms, values or rules when they generate injustice.

To summarize, person-level risk corresponds to the *microsystem* – the characteristics of the individual and how these traits influence their own risk; system-level risk reflects how *health, social, policing, and justice systems* (i.e., exosystem institutions) shape individual risk; and structure-level risk operates at the *macrosystem* level, influencing how institutions function through broader ideologies, histories, and policies such as colonization and systemic racism.

To illustrate how systems and structures can actively produce risk, consider the case of a family member wanting to bring in home-cooked food for a service user during an event of cultural significance (e.g., Ramadan). At the person level, this may be a culturally responsive intervention. It supports the person’s spiritual and cultural needs, promoting a pro-social identity and perceived social support (49), all factors known to reduce risk and recidivism (49–52). Yet, at the system level, such needs are often deprioritized because they fall outside the dominant framing of “criminogenic needs” (53, 54). As a result, rules around food safety, security, and professional boundaries may be enforced without flexibility, even when such flexibility would enhance care and reduce risk. At the structural level, blanket policies that prohibit outside food reflect broader norms that prioritize institutional control over cultural responsiveness (55). These norms often trace back to colonial legacies and assumptions about what counts as legitimate care (56).

From this perspective, risk is no longer an individual attribute, but a product of how systems distribute or withhold care, safety, and opportunity. This reframing shifts forensic mental health from managing “risky individuals” to interrogating and transforming “risky systems.” Any serious attempt to reduce risk must therefore account for system-level vulnerabilities and capacities: how care is coordinated, who is excluded, what assumptions underpin service design, and whether systems can learn and adapt under pressure.

## Building the future of forensic mental health

This paper introduced the *Risk in Systems Framework* to analyze how institutional rules, policies, discourses, and resource flows produce or exacerbate risk. By addressing structural and systemic conditions, the framework redirects attention from individual deficits to the environments that sustain them.

As a conceptual model, the framework now requires empirical testing. Advancing it will involve operationalizing system- and structure-level risk, examining their effects on care pathways and outcomes, and comparing how these dynamics unfold across jurisdictions. This work calls for coordinated international collaborations and access to diverse forensic, social, and policy environments.

Expanding this research agenda also means drawing on disciplines not grounded in individual-level intervention. Public health, urban planning, geography, architecture, and public policy offer conceptual and methodological tools for analyzing how systems distribute care, safety, and opportunity. Architecture and design can illuminate how built environments, particularly in institutional settings, can affect risk of violence and aggression (57, 58). Geography helps explain spatial marginalization (59) while urban planning shows how neighborhood features such as access to green space and opportunities for social connection influence mental health and risk of violence (60, 61). Engineering may help develop technologies that support service delivery (62), whereas public policy can assess the impact of legislation or funding structures on care pathways and outcomes (38, 63). Finally, health economics and health services research can help evaluate the effectiveness, efficiency, and equity of different models of care. While there is growing interest in rethinking models of care in forensic mental health (33, 34), the current knowledge base remains largely descriptive, with little empirical data – especially comparative or cross-jurisdictional (3, 64, 65) – available to guide reform (66). Together, these fields can help expand the forensic mental health research agenda beyond individual-level treatment toward systemic and structural transformation.

However, broadening disciplinary lenses is not enough. Addressing structural risk requires questioning the hierarchies of knowledge embedded within Western academic traditions (53, 67). Dominant models of evidence – assessment tools, randomized controlled trials – often exclude Indigenous and relational knowledge as well as lived experience (56). Upholding Western post-positivist ideals as gold standard for informing policy and practice invalidates alternative ways of knowing and narrows what counts as meaningful data, reliable knowledge, or effective intervention (68–70). In the same vein, forensic mental health research has only minimally engaged with participatory or community-partnered methodologies (71), and even these efforts are often confined to tokenistic consultation. As a result, priorities defined by service users, their chosen support networks, and their communities are often dismissed as anecdotal, unscientific or a “nice to have” (70, 71). Without deeper epistemological shifts – both in what is considered valid knowledge and who are considered legitimate knowers – the field cannot meaningfully study or intervene on the structural risk produced by our societies’ dominant values and ideologies.

Forensic mental health research and systems have reached a tipping point (66, 72). The field cannot continue to focus almost exclusively on the individual and expect meaningful breakthroughs. As Jamie Livingston notes, “striking a balance between doing research that benefits risk assessment/management and public safety while not exacerbating stereotypes about, and stigma for, people with mental health and substance use issues, including those navigating the forensic mental health system, is a principal challenge in the forensic mental health scholarly space” (personal communication, 2025). Recognizing risk as a property of systems rather than individuals shifts responsibility toward the structures that generate vulnerability. This opens the possibility of forensic mental health systems grounded in dignity and shared responsibility.

## Data Availability

The original contributions presented in the study are included in the article/supplementary material, further inquiries can be directed to the corresponding author/s.
